# Osteochondral allograft transplantation for large Hill-Sachs lesions: a retrospective case series with a minimum 2-year follow-up

**DOI:** 10.1186/s13018-019-1366-8

**Published:** 2019-11-07

**Authors:** Hongwu Zhuo, Yangkai Xu, Fugui Zhu, Ling Pan, Jian Li

**Affiliations:** Fuzhou Second Hospital Affiliated to Xiamen University, No.47, Shang Teng Street, Cang Shan District, Fuzhou, 350007 China

**Keywords:** Allograft, Transplantation, Lesion, Resorption, Instability

## Abstract

**Purpose:**

To investigate the clinical outcomes after osteochondral allograft transplantation for large Hill-Sachs lesions.

**Methods:**

Patients who underwent osteochondral allograft transplantation for large Hill-Sachs lesions were identified. Clinical assessment consisted of active range of motion (ROM), American Shoulder and Elbow Surgeons score (ASES), Constant-Murley score, Rowe score, and patient satisfaction rate. Radiographic assessment was performed with CT scan.

**Results:**

Nineteen patients met the inclusion criteria. The mean age was 21.7 years. The mean preoperative size of the Hill-Sachs lesion was 35.70 ± 3.02%. The mean follow-up was 27.8 months. All grafts achieved union at an average of 3.47 months after surgery. At the final follow-up, graft resorption was observed in 43.1% of patients. The average size of residual humeral head articular arc loss was 12.31 ± 2.79%. Significant improvements (*P* < .001) were observed for the active ROM, ASES score, Constant-Murley score, and Rowe score. The overall satisfaction rate was 94.7%. No significant difference was found between the resorption group and the nonresorption group in postoperative clinical outcomes.

**Conclusion:**

Osteochondral allograft transplantation is a useful treatment option for patients with large Hill-Sachs lesions. Although the incidence of graft resorption may be relatively high, the clinical outcomes at a minimum 2-year follow-up are favorable.

**Level of evidence:**

Level IV, case series

## Introduction

Hill-Sachs lesions, which were first described by Hill and Sachs in 1940, typically occur when the softer posterolateral humeral head impacts the harder anteroinferior glenoid rim during traumatic anterior glenohumeral dislocation events [1]. According to the literature, the incidence of Hill-Sachs lesions in first shoulder dislocation is 40 to 90% and nearly 100% in recurrent shoulder dislocation [2]. As these anomalies lead to an articular arc mismatch between the glenoid and humeral head, Hill-Sachs lesions are a well-known risk factor for recurrent shoulder instability and failed shoulder instability surgery [3].

Although small to mid-sized Hill-Sachs lesions can be effectively treated by arthroscopic remplissage, the treatment of large Hill-Sachs lesions involving > 30% of the humeral head remains a significant challenge for orthopedic surgeons [4, 5]. Various methods have been reported for the treatment of large Hill-Sachs lesions, including rotational osteotomy, Latarjet, humeral head reconstruction with an allograft, and arthroplasty [6, 7]. Rotational osteotomy is rarely used due to the variability in the derotation achieved and its reportedly high complication rate. Latarjet fails to address the underlying humeral lesion directly and is a nonanatomic procedure associated with the risk of osteoarthritis, nonunion, and hardware failure. Arthroplasty is not a good option for younger, active cases.

Humeral head reconstruction with osteochondral allograft transplantation is a recently implemented method to restore the native spherical contour of the humeral head and regain shoulder stability [8–14]. A size-matched fresh-frozen humeral or femoral head allograft is placed into the humeral defect and seated flush with the surrounding articular surface, thereby increasing the articular arc of the humeral head as it rotates on the glenoid and preventing engagement. However, due to the inherent limitations of allografts, many orthopedic surgeons have concerns regarding allograft-related complications, including disease transmission, delayed or nonunion, and graft resorption.

To our knowledge, current studies regarding osteochondral allograft transplantation for large Hill-Sachs lesions remain limited [8–14]. The purpose of the present study was to investigate the clinical outcomes in a series of patients who underwent osteochondral allograft transplantation for large Hill-Sachs lesions with a minimum 2-year follow-up. We also wanted to determine the allograft-related complications. We hypothesized that osteochondral allograft transplantation was a useful treatment option and would render favorable clinical outcomes for large Hill-Sachs lesions.

## Materials and methods

### Study design

From January 2013 to January 2017, patients at our institution who underwent osteochondral allograft transplantation for large Hill-Sachs lesions were retrospectively identified. This study received institutional review board approval. All patients provided signed informed consent to allow their clinical and radiologic data to be used for research programs.

The inclusion criteria were (1) patients diagnosed with traumatic recurrent anterior shoulder instability, (2) patients with a large Hill-Sachs lesion > 30% of the humeral head articular surface, (3) patients who underwent osteochondral allograft transplantation, (4) patients who agreed to participate, and (5) patients with complete imaging evaluation data (CT scan at 3, 6, 12, and 24 months after surgery) and a minimum 2-year follow-up. The exclusion criteria were (1) obvious glenoid bone loss > 20%, (2) previous surgery of the ipsilateral shoulder, (3) significant glenohumeral osteoarthritis, or (4) patients who could not cooperate with correct rehabilitation after surgery.

### Preoperative assessment

Data on age, sex, smoking, hand dominance, number of dislocations, age at the first shoulder dislocation, and duration of instability were collected. Clinical assessment consisted of active range of motion (ROM), American Shoulder and Elbow Surgeons (ASES) score, Constant-Murley score, and Rowe score. The forward elevation and external rotation beside the body were measured with a goniometer. The degree of internal rotation was recorded as the highest level the patient’s thumb could reach behind the back. All assessment data were collected by an independent surgeon who was blinded to this study.

**Fig. 1:**
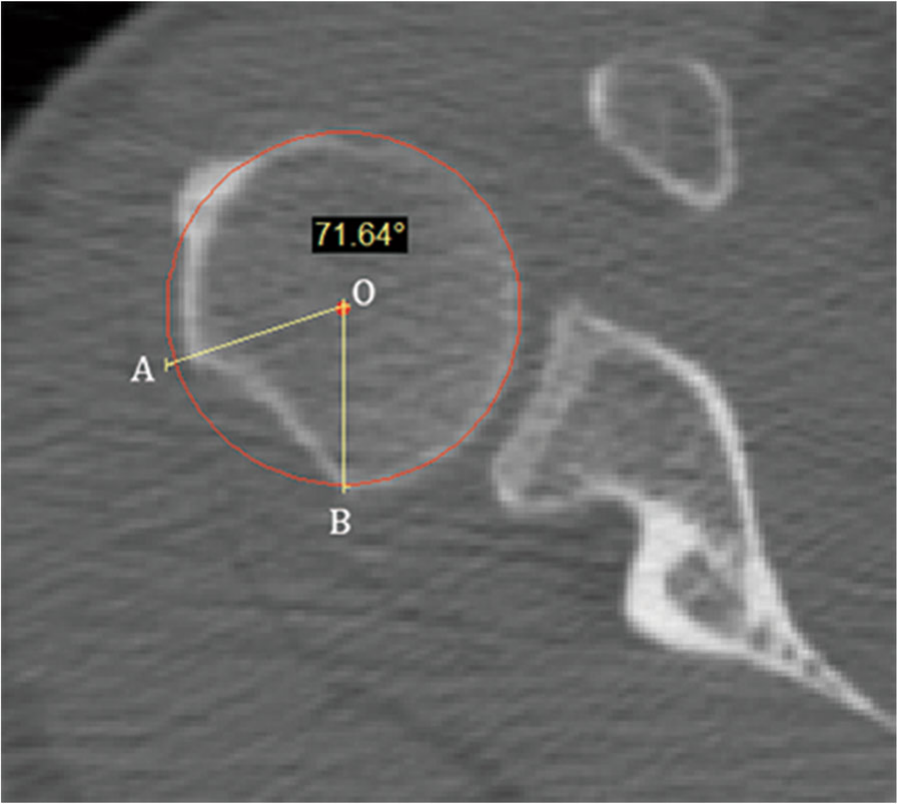
Measurement of the size of the Hill-Sachs lesion in a right shoulder

Before surgery, every patient underwent a standard CT scan to check the bony lesion of the glenoid and the humeral head using an Aquilion 64 CT (Toshiba, Japan) 64-slice scanner. For quantitative evaluation of the glenoid bone lesion, a glenoid en face view was acquired [15]. Patients with glenoid bone loss > 20% were excluded from this study. For quantitative evaluation of the Hill-Sachs lesion, the percentage of humeral head articular arc loss was measured according to the method described by Moroder et al. [16]. The axial image displaying the maximum defect size was chosen. The “best-fit circle” was drawn over the remainder of the borders of the intact articulating surface (Fig. 1). A radial line was drawn from the center of the circle to each limiting edge of the defect, and the angle resulting from the two lines represented the humeral head articular arc loss. The ratio of humeral head articular arc loss to the intact humeral head articular surface (approximately 180°) represented the size of the Hill-Sachs lesion.

### Surgical technique

All surgeries were performed under general anesthesia combined with an interscalene block. The patients were placed in a beach chair position with the shoulder joint placed outside the edge of the operating table to allow the arm to be free into extreme external rotation. Prior to the open intervention, diagnostic arthroscopy was performed to identify other pathologies commonly associated with Hill-Sachs lesions, including Bankart lesions, anterior labral periosteal sleeve avulsion (ALPSA) lesions, humeral avulsion of the glenohumeral ligament (HAGL) lesion, and superior labrum from anterior to posterior (SLAP) tear. In situ repair with suture anchor or simple arthroscopic debridement was performed for the concomitant pathologies.

**Fig. 2:**
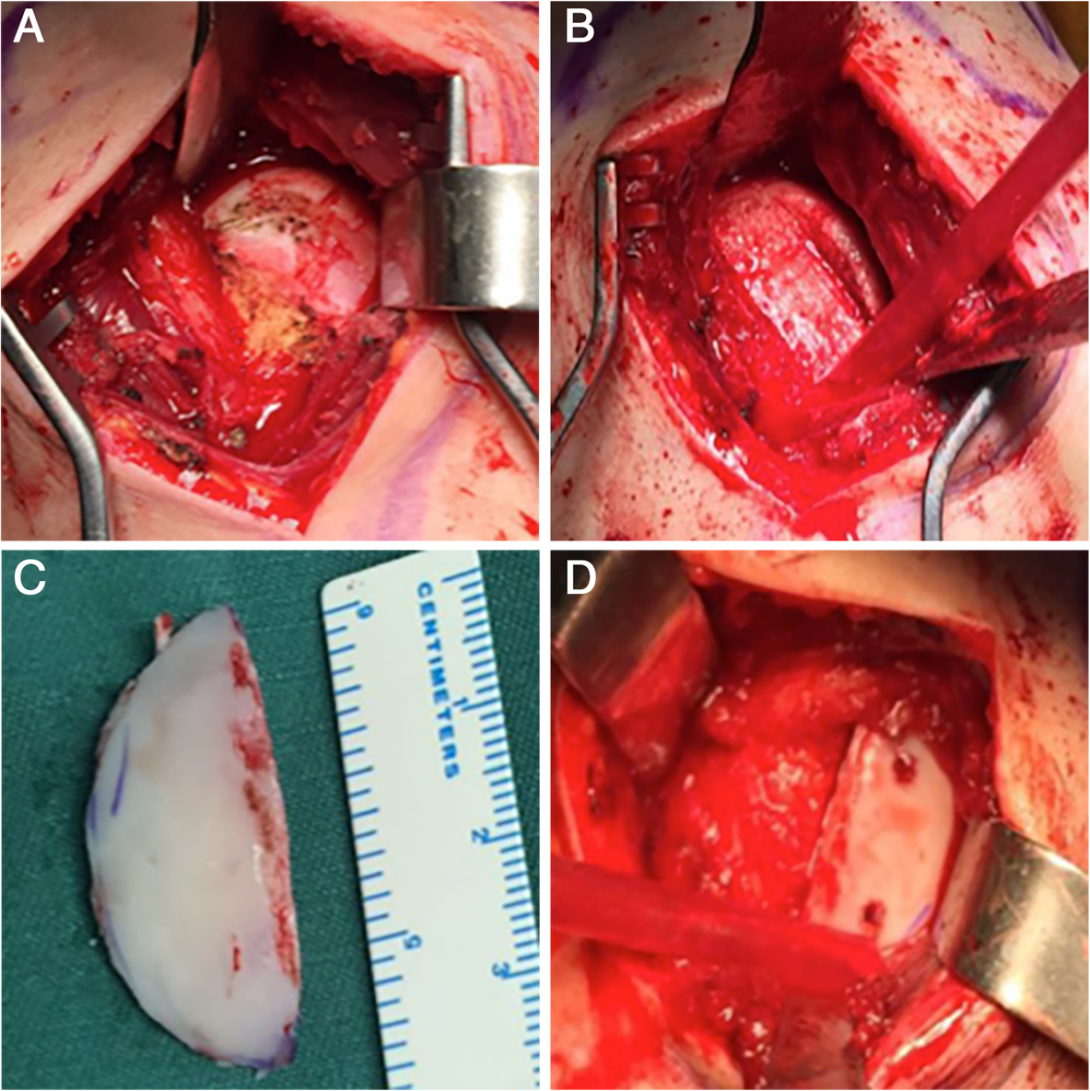
**a** A large Hill-Sachs lesion was exposed. **b** The defect was reshaped into a chevron-type configuration. **c** A wedge of allograft bone was produced on the back table. **d** The graft was secured with two cannulated headless compression screws

For open osteochondral allograft transplantation, a shoulder lateral approach was used. After splitting the raphe between the upper and lower infraspinatus and bringing the arm into extreme external rotation, the Hill-Sachs defect was visualized (Fig. 2a). A sagittal saw was used to smooth and reshape the defect into a chevron-type configuration and to provide a bleeding bone bed onto which the allograft would be fixed (Fig. 2b). The size of the bone bed was measured with a ruler, determining the anterior-posterior, superior-inferior, and medial-lateral dimensions. On the back table, a fresh-frozen allograft femoral head (Lianjie, China) was selected, and a sagittal saw was used to make the bone cuts, producing a wedge of allograft bone approximately 2 mm larger in all dimensions than the measured bone bed (Fig. 2c). The bone graft was then placed into the bone bed and secured with two or three cannulated headless compression screws (Libeier, China) (Fig. 2d).

### Postoperative rehabilitation

After surgery, a shoulder immobilizer was utilized for 8 weeks. The patient was allowed to remove the immobilizer to shower, dress, and perform home exercises. At the first week, the wound was inspected and the patient was allowed to begin pendulum exercises only. At 1 month, active and passive range of motion was initiated under the guidance of an experienced physical therapist. Strengthening started at 3 months. The full return to sport was allowed at 12 months postoperatively.

### Postoperative assessment

Patients were assessed at 1, 3, 6, 12, and 24 months and annually thereafter in the outpatient clinic. The active ROM, ASES score, Constant-Murley score, and Rowe score were used for functional outcome assessment by the same independent surgeon who performed the preoperative assessment. At the final follow-up, the patients were additionally asked about their satisfaction regarding their clinical outcomes (i.e., very satisfied, satisfied, neutral, or not satisfied). The proportion of very satisfied and satisfied patients was defined as the satisfaction rate.

All patients underwent CT scan at 3 months after surgery to check whether the graft achieved union. If the gap between the graft and bone bed disappeared, then it was defined as graft union. Otherwise, if the gap remained visible, then it was defined as graft nonunion, and a CT scan at 3 months later would be required to check again until the graft achieved union.

At both 1 and 2 years after surgery, the patients were asked to take a CT scan to evaluate graft resorption. To assess the influence of graft resorption on the clinical outcomes, the patients were divided into 2 groups. The resorption group consisted of patients in whom graft resorption was observed. The nonresorption group consisted of patients in whom graft resorption was not observed. For patients in the resorption group, the residual humeral head articular arc loss was measured using the same method as previously mentioned.

### Statistical methods

All statistical analyses were performed using SPSS software (IBM-SPSS statistics 23.0; New York, USA). Continuous variables were presented as the mean and standard deviation. A paired-sample *t* test was used to determine the differences between preoperative and postoperative quantitative variables. An independent-sample *t* test was used for between-group comparisons of quantitative variables. Fisher’s exact test was used for between-group comparisons of categorical variables. The significance level was set at 0.05.

## Results

A total of 22 patients were identified, of whom 19 patients met the inclusion criteria and were included in this study (Additional file 1). There were 12 males and 7 females with a mean age of 21.7 years (range, 15–41 years). The dominant side was involved in 13 patients. The mean duration of recurrent instability before surgery was 8.05 years (range, 2–20 years). The mean preoperative size of the Hill-Sachs lesion was 35.70 ± 3.02% (range, 30.2–42.1%). Nineteen concomitant procedures were performed in 17 patients (17 Bankart repairs, 1 superior labrum repair, and 1 superior labrum debridement). The mean duration of follow-up was 27.8 months (range, 24–48 months).

### Radiographic outcomes

**Fig. 3:**
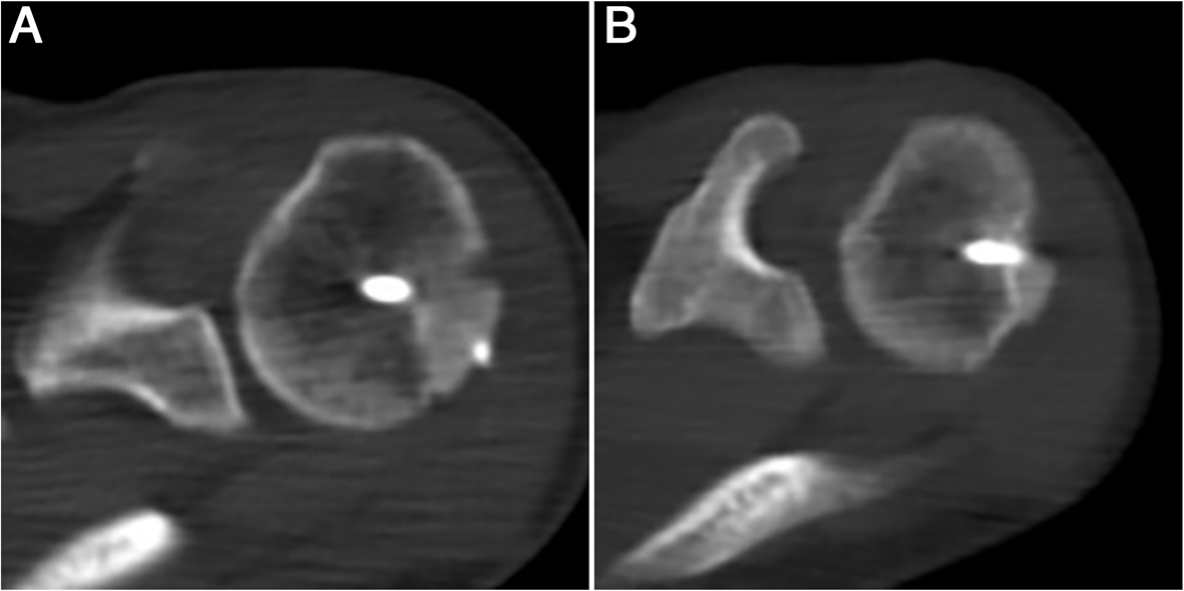
**a** The graft achieved union at 3 months postoperatively. **b** At 1 year after surgery, graft resorption was observed

A postoperative CT scan showed that the graft achieved union at 3 months postoperatively in 16 patients (Fig. 3a) and at 6 months postoperatively in the remaining 3 patients. No graft nonunion was observed in the group. All grafts achieved union at an average of 3.47 months (range, 3–6 months).

At 1 year after surgery, graft resorption was observed in 8 patients (Fig. 3b). The size of residual humeral head articular arc loss was 12.20 ± 2.69%. At 2 years after surgery, graft resorption was still observed only in the 8 patients, and the size of residual humeral head articular arc loss was 12.31 ± 2.79%. No significant difference was found in the size of residual humeral head articular arc loss at 1 and 2 years after surgery (*P* = .565). The incidence of graft resorption was 43.1% (8/19).

### Clinical outcomes

The results of the preoperative apprehension test for all patients were positive. At the final follow-up, the clinical outcomes improved significantly as measured by active ROM, ASES score, Constant-Murley score, and Rowe score (Table 1). No recurrent dislocations or subluxations were detected during the follow-up. The postoperative apprehension signs were all negative. Among the 19 patients, 18 patients were very satisfied or satisfied with their clinical outcomes at the final follow-up. The overall satisfaction rate was 94.7%.

**Table 1:** Comparison of the preoperative and final follow-up clinical outcomes

Variables	Preoperative	At the final follow-up	*P* value
Forward elevation,°	160.3 ± 7.72	170.0 ± 8.16	< .001
External rotation,°	54.7 ± 6.73	61.8 ± 8.85	.011
Internal rotation	T9	T8	.143
ASES score	53.2 ± 6.83	96.9 ± 2.43	< .001
Constant-Murley score	81.1 ± 5.11	88.8 ± 3.48	< .001
Rowe score	23.6 ± 7.22	97.6 ± 2.12	< .001

### Between-group comparisons

**Fig. 4:**
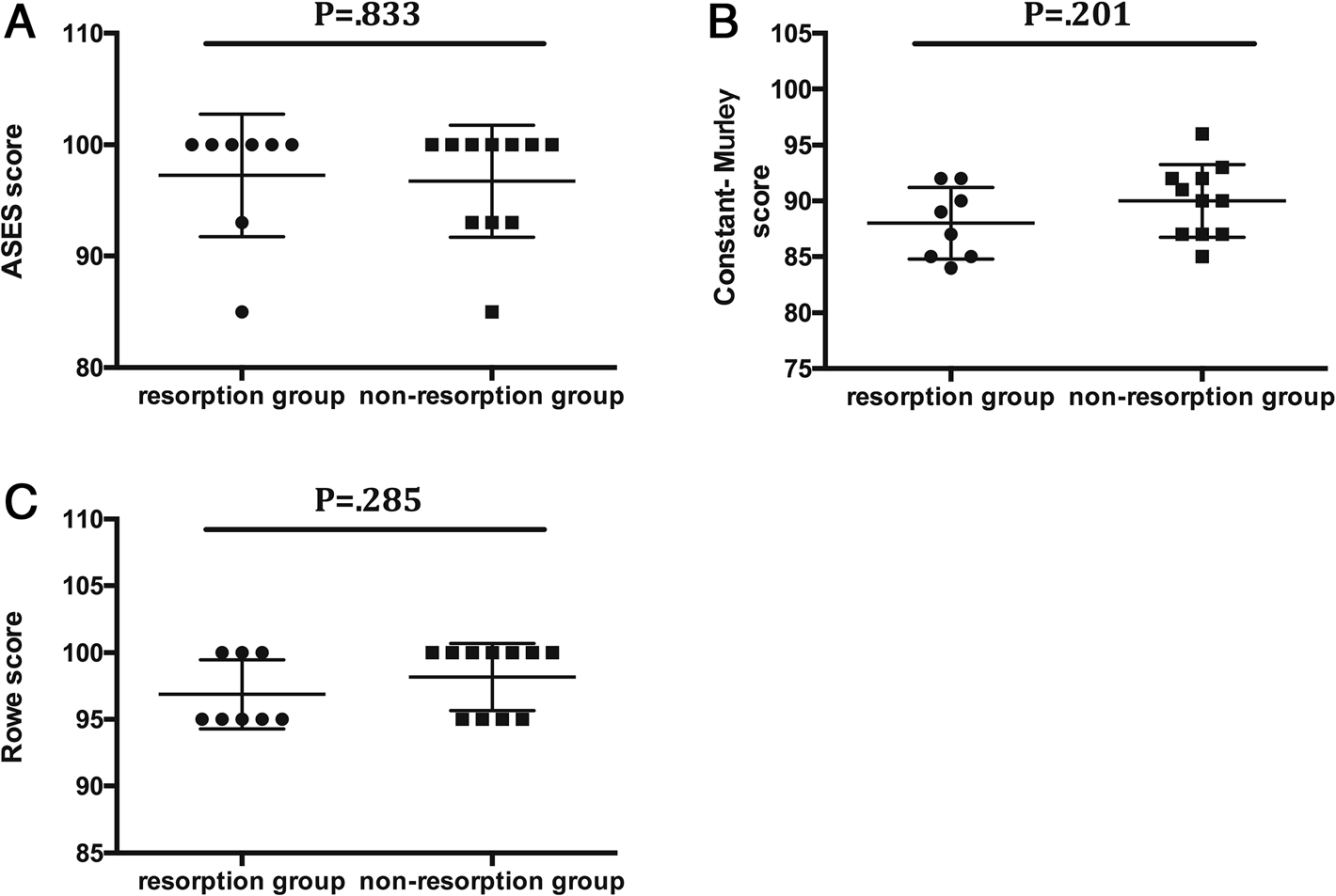
Comparison of final follow-up clinical outcomes between the resorption group and the nonresorption group. **a** The American Shoulder and Elbow Surgeons score (ASES) between the resorption group and the nonresorption group. **b** The Constant-Murley score between the resorption group and the nonresorption group. **c** The Rowe score between the resorption group and the nonresorption group

Three demographic factors showed significant difference between the resorption group and the nonresorption group, including age (*P* = .016), duration of instability (*P* = .021), and preoperative size of the Hill-Sachs lesion (*P* = .001) (Table 2). However, no significant difference was found between the resorption group and the nonresorption group in postoperative ASES score, Constant-Murley score, and Rowe score (Fig. 4a–c).

**Table 2:** Comparison of the demographic factors between the resorption group and the nonresorption group

Variables	Resorption group (*n* = 8)	Nonresorption group (*n* = 11)	*P* value
Age, years	33.6 ± 7.53	25.5 ± 5.73	.016
Sex, male/female	5/3	7/4	1.00
Smoking, yes/no	5/3	4/7	.370
Hand dominance, yes/no	6/2	7/4	1.00
Number of dislocation	12.6 ± 5.95	10.3 ± 4.22	.326
Duration of instability, years	11.0 ± 6.14	5.90 ± 2.21	.021
Preoperative size of lesion, %	38.1 ± 2.22	33.9 ± 2.15	.001

### Complications

During the follow-up, one patient in the resorption group had slight pain in the operative shoulder while participating in overhead sports. The screws were removed at 1 year after surgery, which successfully relieved the patient’s symptoms. No patient in this group developed infection, disease transmission, neurovascular injury, severe stiffness, or significant glenohumeral osteoarthritis.

## Discussion

The main findings of this study were that at a mean follow-up of 27.4 months, osteochondral allograft transplantation for large Hill-Sachs lesions rendered favorable clinical outcomes with a satisfaction rate of 94.7%. All grafts achieved union at an average of 3.47 months (range, 3–6 months). The incidence of graft resorption was 43.1% (8/19). However, there was no significant difference between the resorption group and the nonresorption group in the clinical outcomes.

Although rare, Hill-Sachs lesions still pose a significant challenge to orthopedic surgeons when attempting to restore normal glenohumeral biomechanics and prevent continued subluxation or dislocation events. Generally, the surgical procedures for large Hill-Sachs lesions can be divided into three categories [6–14]: (1) nonanatomy procedures (i.e., humeral rotational osteotomy, Latarjet), (2) anatomy procedures (i.e., humeral head reconstruction with an allograft), and (3) humeral arthroplasty (i.e., partial or total arthroplasty). Compared with the other two categories, the advantages of anatomy procedures include (1) the restoration of the native spherical contour of the humeral head, (2) the maintenance of normal ROM of the joint, (3) a biomechanically stable joint without alteration of the joint kinematics, and (4) the possibility of future prosthetic replacement with no bone stock compromise. On the other hand, the disadvantages include (1) disease transmission, (2) delayed or nonunion, (3) graft resorption, (4) hardware impingement, and (5) lack of reliable long-term clinical outcomes.

Current studies regarding the clinical outcomes after osteochondral allograft transplantation for the management of large Hill-Sachs lesions remain limited primarily to a few case reports or small case series [8–14]. In 2012, Nathan and Parikh reported a case that underwent open osteochondral allograft transplantation for large Hill-Sachs lesions [11]. At the 2-year follow-up, the patient regained full range of motion, reported no further instability, and had radiographic evidence of graft union. Dipaola et al. evaluated the clinical outcomes after osteochondral allograft transplantation for large Hill-Sachs lesions in four patients [12]. At a follow-up of 27.4 months, the average ASES and UCLA shoulder scores were 85.3 and 28.4, respectively. No patients in their study had recurrent instability. The only large case series we found was published by Miniaci and Gish [14]. These authors reported on a cohort of 18 patients who underwent osteoarticular allograft transplantation for large Hill-Sachs lesions. At a follow-up of 50 months, the Western Ontario Shoulder Instability (WOSI) index was improved in all patients, and the average Constant score was 78.5. More than 89% of the patients returned to work, and no patients had recurrent instability. Moreover, 2 of 18 patients had radiographic evidence of graft resorption. However, the weaknesses of the study published by Miniaci and Gish were as follows: (1) the residual humeral head articular arc loss was unknown, and (2) the influence of graft resorption on the clinical outcomes was not analyzed.

Currently, osteochondral allograft transplantation has become a popular technique used to treat a wide spectrum of articular injuries and joint diseases, including glenoid bone loss and osteochondral lesions of the femur and talus [17–19]. Many studies have shown that graft resorption is a common complication. Levy et al. reported on a cohort of 129 patients who underwent osteochondral allograft transplantation for femoral condyle lesions [18]. At 7.2 years after surgery, graft resorption was found in 24% of cases. In another case series, Raikin reported on 15 consecutive patients who underwent osteochondral allograft transplantation for talus lesions [19]. At a follow-up of 44 months, 66.7% (10/15) of cases developed graft resorption. In the present study, we also observed graft resorption in 43.1% of cases at 27.4 months after osteochondral allograft transplantation for large Hill-Sachs lesions.

Although the incidence of graft resorption after osteochondral allograft transplantation is relatively high, many studies suggest that graft resorption has no significant negative influence on the clinical outcomes [19–21]. In a study published by Raikin, although the incidence of graft resorption was up to 66.7%, the American Orthopedic Foot and Ankle Society (AOFAS) score significantly improved from 38 preoperatively to 85 postoperatively (*P* < .05) [19]. Zhu et al. also reported on a cohort of 63 patients who underwent graft transplantation for obvious glenoid loss [20]. At a mean follow-up of 2 years, 90.5% of patients had various degrees of bone resorption after surgery, and 49.2% had major or complete bone graft resorption. However, no significant difference was found in the functional outcomes between the no/minor resorption group and the major/complete resorption group. Similarly, our study also showed no significant difference between the resorption group and the nonresorption group in the clinical outcomes. Nevertheless, the exact reason why graft resorption has no significant negative influence on the clinical outcomes is unknown. We suggest that this finding might be due to the small size of the residual humeral head articular arc loss (12.75 ± 3.80%), which was not large enough to cause a significant influence on the joint stability. As Burkart and De Beer previously found, lesions involving < 20% of the humeral head were rarely of clinical significance [22].

This study has several limitations. First, this study was a retrospective study with all of the inherent limitations of a retrospective study. Second, this study included a limited number of patients due to the low incidence of large Hill-Sachs lesions. To our knowledge, this study cohort is the largest series of patients in the literature who underwent osteochondral allograft transplantation for large Hill-Sachs lesions. Third, the length of the follow-up was relatively short, and longer-term evaluations are required to evaluate the midterm or long-term clinical outcomes after this procedure. Fourth, this study did not compare the index procedure or other surgical interventions with any control group. Last but not least, the influences of concomitant procedures, such as Bankart repair, superior labrum repair, and simple debridement, on the clinical outcomes were not taken into account and warrant further investigation.

## Conclusion

Osteochondral allograft transplantation is a useful treatment option for patients with large Hill-Sachs lesions. Although the incidence of graft resorption may be relatively high, the clinical outcome at a minimum 2-year follow-up is favorable.

## Supplementary information

**Additional file 1.** The patient’s data.

## Data Availability

The datasets used and analyzed during the current study are available from the corresponding author on reasonable request.

## Abbreviations

ALPSA: Anterior labral periosteal sleeve avulsion
ASES: American Shoulder and Elbow Surgeons
HAGL: Humeral avulsion of the glenohumeral ligament
ROM: Range of motion
SLAP: Superior labrum from anterior to posterior
